# Continuous Ultrasound-Guided Lumbar Erector Spinae Plane Block for Thigh Cellulitis Analgesia: A Case Report

**DOI:** 10.7759/cureus.63616

**Published:** 2024-07-01

**Authors:** Rita Lopes Dinis, Ana Sofia Pinto, Ana Faísco

**Affiliations:** 1 Department of Anesthesiology and Pain Therapy, Hospital Professor Doutor Fernando Fonseca, Amadora, PRT

**Keywords:** thigh cellulitis, regional analgesia, lumbar erector spinae, continuous ultrasound-guided block, acute pain

## Abstract

The management of refractory acute medical pain can be challenging, especially if severe and decompensated systemic pathologies contraindicate neuraxial techniques and deep peripheral blocks. In this case report, we propose a continuous ultrasound-guided lumbar erector spinae plane block (ESPB) for multimodal analgesia of thigh cellulitis. The patient was an 80-year-old male, admitted to the intensive care unit due to septic shock originating from cellulitis of the right lower limb, associated with multiorgan dysfunction. To address refractory pain in the thigh, an ultrasound-guided lumbar ESPB at L3 was performed, with the placement of a perineural catheter and administration of 30 mL of 0.5% ropivacaine, followed by 30 mL boluses of 0.375% ropivacaine every six hours with progressive weaning. The patient maintained controlled pain without the need for rescue analgesia. Continuous ultrasound-guided lumbar ESPB is an effective and safe alternative for thigh analgesia in patients with refractory acute medical pain and systemic pathologies that contraindicate other regional techniques.

## Introduction

The erector spinae plane block (ESPB) is an interfascial block useful in various situations due to its ease of administration, safety profile, and excellent level of analgesia [1]. Technically, it is a method in which local anesthetic is injected into the fascial space between the erector spinae muscle and the vertebral transverse process, providing analgesia by covering the dorsal and ventral branches of the spinal nerves [2,3].

ESPB was first described in the literature by Forero et al. in 2016 when it was used to treat chronic neuropathic chest pain [3-9]. Most publications refer to it being performed at the thoracic level, and there is still limited data in the literature on the effectiveness of this block when performed in the lumbar region [2,5,10]. The first description of lumbar ESPB was for postoperative analgesia in the context of hip arthroplasty [7]. Over the last few years, the description of ESPB applications has gradually increased, including uses in the context of acute and chronic pain, not only in the thoracic and lumbar regions but also in the cervical and sacral regions [7,8].

In the surgical context, several studies demonstrate the analgesic efficacy of the lumbar ESPB, leading to a reduced use of opioids [8]. In the literature, applications for perioperative analgesia are described in the context of the hip, lumbosacral spine, lower limb (femur and knee), and thoracic and abdominal surgeries (inguinal hernia, iliac crest autograft, nephrectomy, and Pfannenstiel) [1-7,9,10]. Furthermore, it has been demonstrated that this technique can be used as the main and effective anesthetic method in areas with complex innervation, such as the hip and proximal femur, and also in inguinal hernia surgery. The mechanism can be explained by the dissemination of the local anesthetic to the paravertebral, epidural, and lumbar plexus spaces [7].

With this case report, we intend to demonstrate the usefulness of continuous ultrasound-guided lumbar ESPB for thigh analgesia in a patient with septic shock admitted to the Intensive Care Unit. In this way, we highlight its role in the treatment of refractory acute medical pain outside the operating room in the context of activating the Acute Pain Unit.

## Case presentation

**Figure 1:**
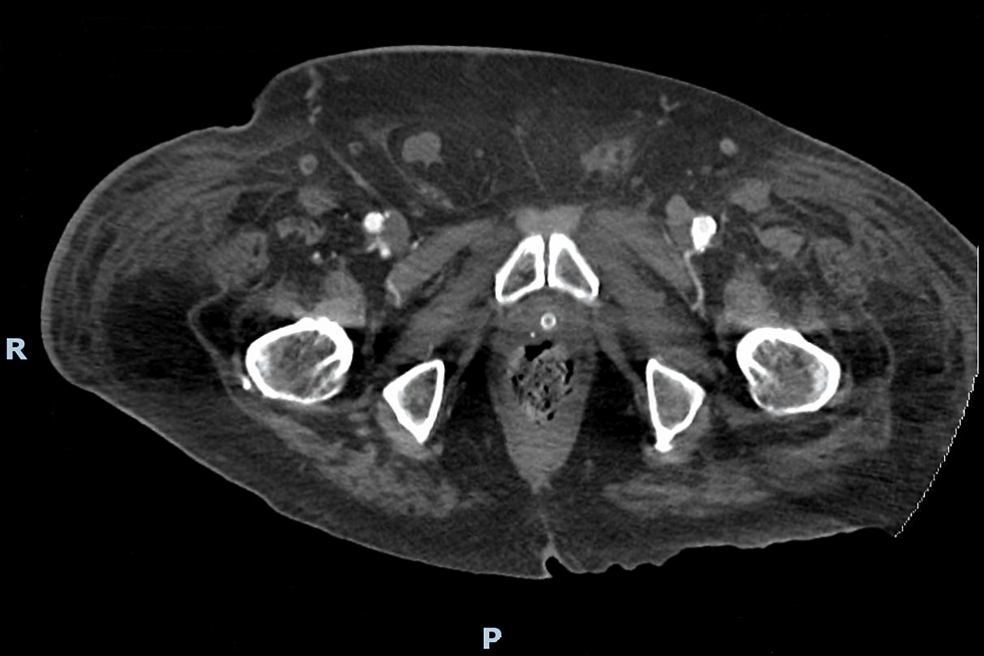
Pelvic computed tomography showing edematous infiltration of the subcutaneous tissues, more exuberant on the right, consistent with cellulitis of the lower limb. R: right; P: posterior

This case involves an 80-year-old male patient with a history of arterial hypertension, permanent atrial fibrillation under anticoagulation, a complete right bundle branch block, heart failure of multifactorial etiology with severe right ventricular dysfunction, asthma, obstructive sleep apnea syndrome under nocturnal continuous positive airway pressure, and peripheral venous insufficiency. The patient was admitted to the Intensive Care Unit due to septic shock originating from cellulitis of the right lower limb (Figure 1), associated with multiorgan dysfunction: cardiovascular (hypotension, heart failure, and hyperlactacidemia requiring vasopressor support with noradrenaline), renal (oliguria), hematological (thrombocytopenia worsening), and hepatic (jaundice and cytocholestasis with hyperbilirubinemia). After an etiological investigation, cellulitis caused by *Streptococcus pyogenes* was identified as concomitant bacteremia, fulfilling the criteria for toxic shock, which led to treatment with targeted antibiotics and immunoglobulin. The right lower limb showed signs of inflammation, with an ample area of blisters and erythema that extended to the proximal region of the thigh, sparing the external face, perineum, and scrotum. Initially, the patient reported pain complaints in the right lower limb only when mobilizing, under fixed analgesia with paracetamol and metamizole magnesium. The complaints then progressed to pain at rest, requiring fentanyl boluses in SOS and, subsequently, fentanyl and ketamine infusions. Due to worsening pain complaints requiring high doses of fentanyl and ketamine, the Acute Pain Unit of the Anesthesiology Department was contacted to optimize multimodal analgesia using regional techniques.

**Figure 2:**
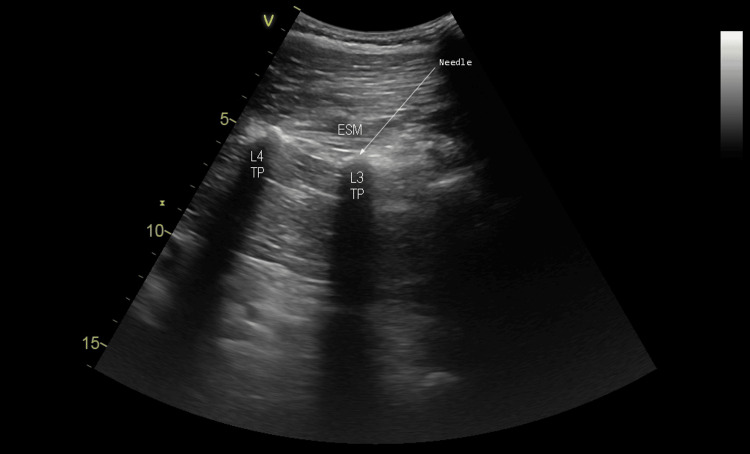
Ultrasound image of the lumbar ESPB. ESM: erector spinae muscle; TP: transverse process; L3: third lumbar vertebra; L4: fourth lumbar vertebra; arrow: needle

The patient reported intense pain during movement, corresponding to a visual analogue scale (VAS) of 8/10 and moderate pain at rest (VAS 5/10), describing the pain in the right thigh as circumferential. After evaluating the patient, several regional analgesia options were considered. We chose not to perform an epidural or lumbar paravertebral block due to worsening thrombocytopenia (minimum platelet value of 68000) and septic shock with active bacteremia. We also excluded peripheral blocks of the lower limb due to the progression of the infection to the proximal region of the thigh. We performed an ultrasound-guided right lumbar ESPB at the L3 level (Figure 2), under standard American Society of Anesthesiologists (ASA) monitoring, using the 4C-RS 1.3-4 MHz convex probe of the GE Vivid iq® ultrasound (Chicago, Illinois).

As our goal was to provide prolonged analgesia for several days, we used the Pajunk® E-Cath® Tsui® perineural catheter kit (Geisingen, Germany) with the echogenic catheter-over-needle system. With the patient in the right lateral decubitus position and under all aseptic conditions, the spinous process of the L3 vertebra was located sonographically using a sagittal orientation of the probe. Then, moving the probe laterally, the transverse process of the same vertebra was identified using a parasagittal approach. We inserted the SonoPlex 21 G x 68 mm with Facet Tip needle and the Indwelling catheter (working length) 18 G x 51 mm in the craniocaudal direction following an in-plane approach, visualizing the needle tip sonographically until it reached the transverse process. Then, a 2 mL bolus of 0.9% NaCl was administered to confirm the correct location, visualizing its deposition between the erector spinae muscle and the transverse process. The needle was then removed and, through the Indwelling catheter, we introduced the E-Catheter 20 G to which the Filter 0.2 µm and the Fixolong were connected. We injected 30 mL of 0.5% ropivacaine through the catheter, sonographically visualizing the craniocaudal spread of the local anesthetic, and fixed the catheter to the skin. The whole technique was uneventful, and the patient reported significant pain relief approximately 10 minutes after the procedure. Subsequent analgesia was prescribed in the form of mandatory intermittent 30 mL boluses of 0.375% ropivacaine every six hours, along with fixed paracetamol and metamizole magnesium. As rescue analgesia, SOS boluses were prescribed through the perineural catheter in the same dose, as well as SOS opioids in the form of fentanyl infusion.

The patient was observed daily by the Acute Pain Unit team and manifested a significant relief of pain in his right thigh, reporting mild pain when moving (VAS 2/10) and the absence of pain at rest (VAS 0/10). No rescue analgesia with opioids or additional boluses through the perineural catheter was necessary, and it was possible to reduce the analgesic regimen to 30 mL of 0.2% ropivacaine every six hours. Subsequently, as the pain remained controlled, the analgesic regimen was further reduced to a rescue 30 mL boluses of 0.2% ropivacaine, together with fixed paracetamol and metamizole magnesium.

On the fifth day of observation, there was a worsening of cellulitis in the right lower limb with progression up to the level of the T7 vertebra. Following consultation with colleagues from the Intensive Care Unit and taking into account that the patient's pain remained controlled, without the need for a rescue bolus of ropivacaine in the last 24 hours, it was decided to remove the perineural catheter, which was an uneventful procedure. The patient was discharged from the Acute Pain Unit that day and remained hospitalized in the Intensive Care Unit, until progressive improvements in organ dysfunction and cellulitis in the right lower limb were observed, resulting in a transfer to the Intermediate Care Unit. The patient provided consent to publish this case report.

## Discussion

The lumbar ESPB is relatively recent and represents a new horizon for regional analgesia and pain management, improving the quality of analgesia when added to a multimodal scheme [7]. The deposition of local anesthetic in the fascial plane of the erector spinae causes its extensive craniocaudal spread in that plane and possibly also at the paravertebral and epidural levels, blocking the dorsal, ventral, and communicating branches of the spinal nerves [1]. The ultrasound-guided technique has been described in the literature as an effective analgesic method in various situations, such as the perioperative period and chronic pain, despite presenting a much lower number of publications when compared to the thoracic block. Furthermore, reports of its application in acute pain scenarios are still scarce, especially in the case of continuous block through the placement of a perineural catheter.

In this case, support from the Acute Pain Unit was requested due to the patient’s complex clinical scenario, characterized by prolonged hospitalization in the Intensive Care Unit due to septic shock originating from cellulitis of the lower limb and presenting acute pain at the level of the thigh refractory to opioids and multimodal analgesia. We considered various pain management strategies, including deep neuraxial and nerve blocks such as lumbar epidural and lumbar paravertebral. Since the patient was presenting bacteremia and hematological dysfunction associated with septic shock, manifesting as worsening thrombocytopenia (minimum platelet value of 68000), we decided not to perform deep techniques due to the risk of infectious complications and deep hematomas, particularly at the neuraxial level.

We also considered performing superficial and peripheral nerve blocks at the lower limb level, such as femoral and fascia iliaca, although we excluded this possibility due to the infectious risk at the puncture site, as the patient had a worsening infectious process extending to the proximal region of the thigh. Therefore, we chose to perform an ultrasound-guided lumbar ESPB at the L3 level, placing a perineural catheter that allowed prolonged analgesia. We found that the initial dose of 30 mL of 0.5% ropivacaine was effective in significantly relieving pain complaints, as well as the following analgesic regimen with 30 mL of 0.375% ropivacaine every six hours, with subsequent weaning to 0.2% ropivacaine. While the perineural catheter was in place, the patient always maintained pain control without the need for a rescue bolus of opioids or a local anesthetic. The catheter ended up being removed due to the progression of the infection to the thoracic level. The lumbar ESPB was uneventful, but potential complications of the technique include infection, bleeding from the puncture site, and nerve injury. Due to this, it is very important to monitor inflammatory signs, hematomas, and neurological deficits and be prepared to perform drainage or increase antibiotic coverage. Furthermore, the analgesia was performed with high-volume, mandatory intermittent boluses, so it is imperative to monitor signs of local anesthetic systemic toxicity. If a clinical scenario compatible with this condition arises, it is of crucial importance to follow the treatment protocol for this anesthetic emergency.

Two reports on the use of a catheter in the lumbar erector spinae plane in knee surgery described successful results [7]. Furthermore, Kinjo and Schultz [4] described clinical cases in which catheter placement in the lumbar erector spinae plane provided excellent postoperative analgesia in patients undergoing total hip arthroplasty revision. However, there is still no consensus on the best administration regime between bolus and continuous infusion nor the perfusion rate in the catheter, but the maximum daily dose must be kept in mind. Dispersion of local anesthetic between dermatomes T12 and S1 has been documented when a volume between 30 and 40 mL is administered at the lumbar level [7].

In the context of chronic pain, Durmus et al. [8] demonstrated that a single-shot lumbar ESPB produces a significant effect in reducing the intensity of chronic low back pain, with relief of radicular symptoms in patients with lumbar disc herniation. Kokar et al. [2] also demonstrated the usefulness of this technique in lumbar radicular pain, which may be preferred as an alternative to the peri-radicular injection, as it reduces the risk of complications since it is a superficial injection technique. Additionally, this block has been applied after herniorrhaphy and in a variety of painful syndromes, including complex regional pain syndrome, radiculopathy, myofascial pain, and chronic cancer pain, as seen in several case reports [7,8]. Alici et al. [11] described the application of high-volume single-shot lumbar ESPB (40 mL) in the treatment of postherpetic neuralgia, manifested by pain in several dermatomes of the lower limb. This clinical case suggested that this procedure can provide lower limb analgesia and that the lumbar and sacral plexuses can be blocked if a high volume of local anesthetic is used.

Even though the literature regarding the application of lumbar ESPB is scarce, the majority of the publications document its application as single-shot ultrasound-guided in the perioperative setting, in a variety of surgical interventions. Mujahid et al. [1] reported its effective use as an alternative analgesic method after hip hemiarthroplasty, in a patient with multiple pathologies and not a candidate for the neuraxial technique. Townsend et al. [9] investigated the effectiveness of preoperative block in reducing opioid consumption in the postoperative period of total hip arthroplasty performed under spinal anesthesia, observing benefits in reducing opioid use in the first eight postoperative hours. On the other hand, Ahiskalioglu et al. [5] reported the use of this block combined with low-dose sedoanalgesia as the main, effective, and safe anesthetic method in high-risk and elderly patients undergoing hip surgery.

In this study, magnetic resonance imaging demonstrated that local anesthetic spreads to the L2-L5 nerve roots and the epidural space. Furthermore, a high volume spreads to the lumbar plexus and, therefore, this technique can function as a lumbar plexus block, as already suggested in other publications [7]. Additionally, Balaban et al. [3] demonstrated the feasibility of the technique in the pediatric age group, providing effective postoperative analgesia in femoral fracture surgery when added to multimodal analgesia. Langnas et al. [10] reported its preoperative performance for postoperative analgesia of above-knee amputation, allowing adequate postoperative pain control. Finally, Jin et al. [6] described that pre-incision bilateral block provides effective analgesia in the perioperative period of lumbar laminoplasty, reducing the need for anesthetic and analgesic drugs.

In addition to the existing descriptions of the potential use of the lumbar ESPB, our case study demonstrated the effectiveness of the continuous technique in the treatment of acute medical refractory pain of the thigh due to cellulitis of the lower limb, in a patient admitted to the Intensive Care Unit with severe systemic pathologies that limited regional analgesia options.

## Conclusions

According to this clinical case, the continuous ultrasound-guided lumbar ESPB is an effective and safe alternative for analgesia of thigh cellulitis, providing pain control when added to multimodal analgesia. The lumbar ESPB could be a valuable option for thigh analgesia in different clinical scenarios, especially when deep neuraxial and nerve blocks cannot be performed safely. In the future, randomized controlled clinical trials will determine the efficacy of the lumbar ESPB, and other studies are required to confirm the safe and effective dose of local anesthetic for continuous blocks.

## References

[1] Mujahid OM, Dey S, Nagalikar S, Arora P, Dey CK (2021). Ultrasound-guided lumbar ESP block for post-operative analgesia as an alternative mode of analgesia in hip arthroplasty with multiple systemic issues: a case report. Ain-Shams J Anesthesiol.

[2] Kokar S, Ertaş A, Mercan Ö, Yıldırım FG, Taştan ÖA, Akgün K (2022). The lumbar erector spinae plane block: a cadaveric study. Turk J Med Sci.

[3] Balaban O, Koçulu R, Aydın T (2019). Ultrasound-guided lumbar erector spinae plane block for postoperative analgesia in femur fracture: a pediatric case report. Cureus.

[4] Kinjo S, Schultz A (2019). Continuous lumbar erector spinae plane block for postoperative pain management in revision hip surgery: a case report (Article in Portuguese). Braz J Anesthesiol.

[5] Ahiskalioglu A, Tulgar S, Celik M, Ozer Z, Alici HA, Aydin ME (2020). Lumbar erector spinae plane block as a main anesthetic method for hip surgery in high risk elderly patients: initial experience with a magnetic resonance imaging. Eurasian J Med.

[6] Jin Y, Zhao S, Cai J (2020). Efficacy of ultrasound-guided erector spinae plane block for perioperative pain control and short-term outcomes in lumbar laminoplasty. [PREPRINT] MedRxiv.

[7] Tulgar S, Aydin ME, Ahiskalioglu A, De Cassai A, Gurkan Y (2020). Anesthetic techniques: focus on lumbar erector spinae plane block. Local Reg Anesth.

[8] Durmus IE, Surucu S, Muz A, Takmaz SA (2023). The effectiveness of erector spinae plane block in patients with chronic low back pain. Eur Rev Med Pharmacol Sci.

[9] Townsend D, Siddique N, Kimura A (2022). Lumbar erector spinae plane block for total hip arthroplasty comparing 24-hour opioid requirements: a randomized controlled study. Anesthesiol Res Pract.

[10] Langnas E, Gray A, Braehler M (2021). Erector spinae plane block for postoperative analgesia for above-the-knee amputation: a case report. [PREPRINT] Research Square.

[11] Alici HA, Ahiskalioglu A, Aydin ME, Ahiskalioglu EO, Celik M (2019). High volume single injection lumbar erector spinae plane block provides effective analgesia for lower extremity herpes zoster. J Clin Anesth.

